# Depression, emotional eating and long-term weight changes: a population-based prospective study

**DOI:** 10.1186/s12966-019-0791-8

**Published:** 2019-03-20

**Authors:** Hanna Konttinen, Tatjana van Strien, Satu Männistö, Pekka Jousilahti, Ari Haukkala

**Affiliations:** 1 Department of Food and Nutrition, P.O. Box 66, 00014 University of Helsinki, Helsinki, Finland; 2 Faculty of Social Sciences, P.O. Box 54, 00014 University of Helsinki, Helsinki, Finland; 3 Behavioural Science Institute, Radboud University Nijmegen, P.O. Box 9104, 6500 HE, Nijmegen, The Netherlands; 4 Department of Health Sciences, Faculty of Science, Amsterdam Public Health Research Institute, Vrije Universiteit Amsterdam, 1081 HV, Amsterdam, The Netherlands; 5 Department of Public Health Solutions, National Institute for Health and Welfare, P.O. Box 30, 00271 Helsinki, Finland

**Keywords:** Depressive symptoms, Emotional eating, Three-factor eating questionnaire, Physical activity, Sleep, Weight gain, Obesity, Longitudinal study, Moderation, Mediation

## Abstract

**Background:**

Emotional eating (i.e. eating in response to negative emotions) has been suggested to be one mechanism linking depression and subsequent development of obesity. However, studies have rarely examined this mediation effect in a prospective setting and its dependence on other factors linked to stress and its management. We used a population-based prospective cohort of adults and aimed to examine 1) whether emotional eating mediated the associations between depression and 7-year change in body mass index (BMI) and waist circumference (WC), and 2) whether gender, age, night sleep duration or physical activity moderated these associations.

**Methods:**

Participants were Finnish 25- to 74-year-olds who attended the DILGOM study at baseline in 2007 and follow-up in 2014. At baseline (*n* = 5024), height, weight and WC were measured in a health examination. At follow-up (*n* = 3735), height, weight and WC were based on measured or self-reported information. Depression (Center for Epidemiological Studies - Depression Scale), emotional eating (Three-Factor Eating Questionnaire-R18), physical activity and night sleep duration were self-reported. Age- and gender-adjusted structural equation models with full information maximum likelihood estimator were used in the analyses.

**Results:**

Depression and emotional eating were positively associated and they both predicted higher 7-year increase in BMI (*R*^2^ = 0.048) and WC (*R*^2^ = 0.045). The effects of depression on change in BMI and WC were mediated by emotional eating. Night sleep duration moderated the associations of emotional eating, while age moderated the associations of depression. More specifically, emotional eating predicted higher BMI (*P* = 0.007 for the interaction) and WC (*P* = 0.026, respectively) gain in shorter sleepers (7 h or less), but not in longer sleepers (9 h or more). Depression predicted higher BMI (*P* < 0.001 for the interaction) and WC (*P* = 0.065, respectively) increase in younger participants, but not in older participants.

**Conclusions:**

Our findings offer support for the hypothesis that emotional eating is one behavioural mechanism between depression and development of obesity and abdominal obesity. Moreover, adults with a combination of shorter night sleep duration and higher emotional eating may be particularly vulnerable to weight gain. Future research should examine the clinical significance of our observations by tailoring weight management programs according to these characteristics.

**Electronic supplementary material:**

The online version of this article (10.1186/s12966-019-0791-8) contains supplementary material, which is available to authorized users.

## Background

It has been estimated that worldwide over 300 million people suffer from depression and over 650 million are affected by obesity [1, 2]. The consequences of these conditions in terms of lost health, functioning and quality of life are huge – depression and obesity are both related to an elevated risk of developing several chronic diseases and depression is a major contributor to suicide deaths [1, 2]. There is thus a critical need to develop interventions that are effective in reducing the occurrence of both conditions. Numerous studies have demonstrated that depression and obesity often occur together and are bi-directionally associated over time [3, 4]. In an exploration of possible underlying mechanisms linking depression and obesity, a population-based cross-sectional study showed the link to be mediated by emotional eating [5, 6]. Emotional eating refers to a tendency to eat in response to negative emotions (e.g. depression, anxiety, stress) with the chosen foods being primarily energy-dense and palatable ones [6–8]. It can be caused by various mechanisms, such as using eating to cope with negative emotions or confusing internal states of hunger and satiety with physiological changes associated with emotions [9]. Using the 7-year follow-up data of the same population-based sample, the present study assessed whether emotional eating also acts as a mediator between depression and subsequent weight gain, and whether such a mediation effect is dependent on other factors, including gender, night sleep duration and physical activity. A more detailed knowledge of these factors may point out novel targets for improved obesity and depression interventions to decrease the global burden of disease and increase individual well-being.

Depression (depression-melancholia) is typically characterized by loss of appetite and subsequent weight loss, but there exists also a depression subtype that is characterized by the a-typical vegetative symptom of increased appetite and weight gain [10–12]. Emotional eating has been considered a marker of this a-typical depression subtype, because it shares with this depression subtype the a-typical feature of increased appetite in response to distress [13, 14]. The depression – obesity link may therefore be mediated by emotional eating, for which there was indeed support in various cross-sectional studies for both genders [5, 6, 15, 16] and for women [17]. To date, studies have rarely examined the links between depression, emotional eating and weight gain in a prospective setting. As an exception, a 5-year study in Dutch parents [18] and an 18-year study in mid-life US adults [19] demonstrated that emotional eating acted as a mediator between depression and BMI gain or obesity development particularly in women. With the evidence from the above studies regarding gender being partly mixed, it remains inconclusive whether the mediation effect of emotional eating varies across women and men. Gender was therefore one of the moderators tested in the present prospective study.

The mediation effect of emotional eating between depression and weight gain may also depend on physical activity and sleep duration, though to our best knowledge their moderating effects in this context have not been directly tested before. Both factors have been linked to stress and its management, with exercise being a treatment for depression and anxiety disorders [20–22] and short sleep duration being associated with psychological stress [23, 24]. Higher physical activity has also been associated with lower emotional eating [25, 26]. Accordingly, it has been proposed that increasing physical activity could be a viable strategy to reduce excessive intake of high-fat and -sugar foods under negative emotional states [27] and extending sleep duration could have comparable effects [28]. Exercise could thus attenuate the effects of depression and emotional eating on weight gain via improvements in emotion regulation. In contrast, short sleep duration might strengthen their effects on weight gain – i.e. reduced sleep can be seen as a stressor itself and a marker of perceived stress [29, 30] and evidence is emerging that it interferes with emotion regulation [31]. In support of this, findings from a laboratory study of 64 women suggested that short sleep duration (less than 7 h per night) may act as a stressor and lead to elevated snack intake in those prone to emotional eating [32].

A few observational studies have also found that sleep duration and physical activity moderated the emotional eating – weight gain association. Van Strien and Koenders [29] studied a sample of Dutch employees and observed that women with a combination of short sleep duration and high emotional eating experienced the greatest increases in body mass index (BMI) over 2 years. A similar pattern of findings was reported by Chaput et al. [33] in a sample of French Canadian adults with 6-year follow-up and information on disinhibited eating behaviour (i.e. tendency to overeat in response to food or emotional cues). Moreover, emotional eating was less strongly associated with BMI and its gain in participants with high physical activity than in those with low physical activity in the Dutch employee sample [34] and in a Swiss population survey [26]. However, it is important to explore whether these findings can be replicated and extended using an independent sample of adults with long-term follow-up as well as information on symptoms of depression and change in abdominal obesity.

In the present study, we used a large population-based 7-year prospective cohort of adults to increase our knowledge on the interplay between depression, emotional eating and weight changes in the context of gender, night sleep duration and physical activity patterns. Because of the large age range (between 25 and 74 years at baseline) in this sample, we were also interested in the possible moderating effects of age. More specifically, our aims were to examine 1) whether emotional eating mediated the associations between symptoms of depression and 7-year change in BMI and waist circumference (WC), and 2) whether gender, age, night sleep duration or physical activity moderated these associations.

## Methods

### Participants and procedure

Participants were 25- to 74-year-old Finnish men and women who attended the baseline (*n* = 5024) and follow-up (*n* = 3735) phases of the DIetary, Lifestyle and Genetic determinants of Obesity and Metabolic syndrome (DILGOM) study (for a participant flow chart, see [35]). The baseline phase was conducted in 2007 as a part of the FINRISK 2007 study in which a random sample of 10,000 people, stratified by 10-year age groups and gender, was drawn from the Finnish population register in five large study areas [36]. All participants who attended the FINRISK 2007 study (*n* = 6258, response rate = 63%) in January–March were invited to the DILGOM 2007 study (*n* = 5024, response rate = 80%) conducted in April–June. The baseline phase contained a health examination (including measurements on height, weight and WC) at a study center and several self-administered questionnaires completed either during the visit or at home. All baseline participants alive at the end of the year 2013 received an invitation to the follow-up phase conducted in April–June 2014 (*n* = 3735, response rate = 82%). The data collection was carried out in two groups: 1) participants who lived in the areas of Turku and Loimaa and in the cities of Helsinki and Vantaa were invited to a similar health examination to the one at baseline (*n* = 1312); 2) participants who lived in the other three study areas (North Karelia, North Savo, Oulu) received a mail-back questionnaire and self-reported their current weight and height (*n* = 2423). They also measured their WC themselves, with a measurement tape that was sent to them together with detailed measurement instructions. Participants who did not attend the follow-up phase were more often men (χ^2^ = 7.22, df = 1, *P* = 0.007) and tended to be younger (F(1, 5022) = 13.83, *P* < 0.001, η^2^ = 0.003) and have higher BMI and WC (F(1, 5015) = 26.56, *P* < 0.001, η^2^ = 0.005 and F(1, 4992) = 30.88, *P* < 0.001, η^2^ = 0.006, respectively) at baseline than follow-up participants, but these mean differences were small in size. There were no statistically significant differences between these two groups of participants in terms of baseline education (F(1, 4983) = 3.68, *P* = 0.055, η^2^ = 0.001), depression (F(1, 4727) = 3.70, P = 0.055, η^2^ = 0.001) or emotional eating (F(1, 4853) = 0.60, *P* = 0.438, η^2^ = 0.000).

The research protocols of the DILGOM baseline and follow-up studies were designed and conducted in accordance with the guidelines of the Declaration of Helsinki and have been approved by the Ethics Committee of Helsinki and Uusimaa Hospital District (decision numbers 229/E0/2006 and 332/13/03/00/2013, respectively). In addition, written informed consent was obtained from all participants.

### Outcome variables

#### BMI and WC

Trained research nurses measured participant’s height, weight and WC by using standardized international protocols [37] at baseline and follow-up. Weight was measured to the nearest 0.1 kg, height to the nearest 0.1 cm and WC to the nearest 0.5 cm. All measurements were made in a standing position in light clothing and without shoes. WC was measured at a level midway between the lower rib margin and iliac crest. At baseline, weight and height measurements were available for 5017 (99.9%) participants to calculate BMI (kg/m^2^), while WC measurement was available for 4994 (99.4%) participants. At follow-up, BMI and WC were based on measured (*n* = 1310 and 1305, respectively) or self-reported (*n* = 2352 and 2288, respectively) information. In a recent validation study conducted in a subset of DILGOM participants, the mean differences between self-reported and nurse-measured height, weight and WC were small and the intra-class correlations were 0.95 or greater in both genders [38]. Respondents with measured and self-reported anthropometric data at follow-up were therefore included in this study.

### Predictor variables

#### Depression

The 20-item Center for Epidemiological Studies - Depression (CES-D) Scale [39] was used to measure depressive symptoms at baseline. The scale is designed to measure depressive symptomatology in the general population, and it has been found to be adequately related to clinical ratings of depression [40]. For each item, respondents indicate how often they have felt in the described way during the past week using a four-point scale (from 0 “rarely or none of the time” to 3 “almost all of the time”). A meta-analysis of 28 studies examining the structure of the CES-D scale concluded that the proposed four-factor structure (negative affect, somatic and retarded activity, lack of positive affect, interpersonal difficulties) best described the scale [41]. In line with this and our previous cross-sectional study [5], we modelled depression as a latent factor with four indicators where each indicator was the mean of the items belonging to the respective original factor. It is noteworthy that the CES-D scale contains one item on loss of appetite (“I did not feel like eating; my appetite was poor”), while there is no corresponding item on increased appetite. We decided to exclude the loss of appetite item from the present analyses, because it represents an unbalanced measurement of appetite change with potentially biasing the measurement towards depression subtype characterized by decreased appetite and weight loss. Thus, somatic and retarded activity indicator variable was calculated based on 6 items instead of 7 items.

#### Emotional eating

Emotional eating at baseline was assessed by using the emotional eating scale of the 18-item Three-Factor Eating Questionnaire (TFEQ-R18) [42]. Karlsson et al. [42] developed the TFEQ-R18 on the basis of a factor analysis of the original 51-item TFEQ in the Swedish Obese Subjects study and it has been found to be valid in the general population [43, 44]. The scale contains three items that are all rated on a four-point scale (from 1″ does not describe me at all″ to 4″ describes me exactly″): 1) When I feel anxious, I find myself eating, 2) When I feel blue I often overeat, and 3) When I feel lonely, I console myself by eating. In line with our previous cross-sectional study [5], emotional eating was modelled as a latent factor with the three items as indicators.

### Moderators and covariates

#### Night sleep duration and physical activity

Night sleep duration at baseline was assessed with the following question “How many hours per night do you usually sleep?”. The item was treated as a continuous variable in the analyses. Physical activity at baseline was measured with the International Physical Activity Questionnaire - Short Form (IPAQ-SF) [45]. It asks respondents to report their physical activity during the past 7 days across a comprehensive set of domains (leisure time, work, transport, domestic work and gardening) and three intensity levels (vigorous activities, moderate activities and walking). The data were scored according to the IPAQ manual and a combined total physical activity score (minutes per week) was used on a continuous scale in the main analyses. We repeated the analyses with vigorous physical activity score (minutes per week), but it should be noted that 41.6% of participants had not engaged in any vigorous activities during the past week.

#### Age and gender

Baseline age was treated as a continuous variable (years) and gender as a dichotomous variable (1 = men, 2 = women) in the analyses.

### Statistical methods

We used structural equation modelling (SEM) to test the hypothesized mediation models between depression, emotional eating and 7-year change in adiposity indicators. Depression and emotional eating were modelled as latent factors because ignoring measurement error in predictors can lead to biased regression coefficients and latent variables allow taking measurement error into account [46]. The analyses were conducted in three steps. Firstly, confirmatory factor analysis with two latent factors (depression and emotional eating) was used to test whether the four depression indicators and the three emotional eating indicators loaded on separate factors. Secondly, the hypothesized mediation models with baseline age and gender as covariates were estimated separately for change in BMI and WC – change modelled by regressing the measurement at follow-up on the baseline measurement. The absence of an interaction between exposure (i.e. depression latent factor) and mediator (i.e. emotional eating latent factor) in both models allowed us to apply the SEM approach to mediation analysis (β = 0.12, SE = 0.07, *P* = 0.080 and β = 0.04, SE = 0.07, *P* = 0.585 for the interaction in the model for BMI and WC, respectively) [46, 47]. The results were reported as the total, direct and indirect effects (i.e. regression coefficients and bias-corrected bootstrap 95% confidence intervals) of depression and emotional eating. The reported indirect effect reflects how much of the association between depression and change in adiposity indicator is explained by emotional eating [48]. The total effect represents the relationship between depression and change in adiposity indicator before adjustment for emotional eating. Thirdly, the moderator effects of gender, age, night sleep duration and physical activity were examined in a separate set of models by adding a moderator (in the case of sleep duration and physical activity) and interaction terms (moderator × emotional eating, moderator × depression) as predictors, and testing the significance of these interactions (Mplus code was obtained from Stride et al. [49] – model 59 with X and M as latent variables).

Full Information Maximum Likelihood (FIML) was used as an estimator, which allows estimation with missing data [50, 51]. It does not impute missing values, but estimates parameters directly using all the observed data. Model fit was evaluated by utilizing Chi-Square statistic, Standardized Root Mean Square Residual (SRMR), Tucker-Lewis Index (TLI), Comparative Fit Index (CFI), and Root Mean Square Error of Approximation (RMSEA). As proposed by Hu and Bentler [52], TLI and CFI values ≥0.95, SRMR values ≤0.08, and RMSEA values ≤0.06 were defined to indicate an adequate fit for the data. Descriptive statistics were derived from IBM SPSS Statistics for Windows, Version 24.0 (IBM Corp., Armonk, NY), while all other analyses were performed with Mplus Version 8 (Muthén & Muthén, Los Angeles, CA).

## Results

Descriptive characteristics of the DILGOM participants at baseline in 2007 and follow-up in 2014 are displayed in Table 1. Participants’ WC mostly increased during the 7-year study period with a mean increase of 2.3 ± 6.4 cm in men and 2.1 ± 7.5 cm in women, while their BMI remained rather stable (mean change of 0.0 ± 2.0 kg/m^2^ in men and 0.2 ± 2.3 kg/m^2^ in women). Using the definition of weight maintenance suggested by Stevens et al. [53], a quarter of participants (26% of men and 25% of women) could be defined as weight losers (lost ≥3% of their initial weight) and one-third of them (33% of men and 39% of women) could be defined as weight gainers (gained ≥3% of their initial weight). Change in BMI (F(2, 3657) = 99.88, *P* < 0.001, η^2^ = 0.052) and WC (F(2, 3571) = 59.70, *P* < 0.001, η^2^ = 0.032) varied across age groups with 25–39-year-olds (0.6 ± 2.4 kg/m^2^ for BMI and 3.6 ± 7.6 cm for WC) and 40–59-year-olds (0.4 ± 1.9 kg/m^2^ and 2.9 ± 6.4 cm, respectively) showing greater mean increases than 60–74-year-olds (− 0.5 ± 2.1 kg/m^2^ and 0.5 ± 7.1 cm, respectively). Average night sleep duration was 7.3 h with 18.5% of participants sleeping less than 7 h per night. The respective percentages for 7 h, 8 h, and 9 h or more were 38.2, 34.9, and 8.4%. On average, participants spent 12.4 h (median 9.0 h) per week in physical activity of vigorous or moderate intensity or walking. For vigorous physical activity, the mean and median values were 2.8 h and 1.0 h per week. Pearson’s correlations between the main study variables can be found from Additional file 1.

**Table 1:** Descriptive characteristics of the Finnish DILGOM participants at baseline in 2007 and follow-up in 2014

	All participants	Participants who attended baseline and follow-up phases	
	Year 2007	Year 2007	Year 2014
Number of participants^a^	4729–5024	3554–3735	3593–3735
Age (years)	52.6 ± 13.5^b^	53.0 ± 13.0	60.0 ± 13.0
Gender			
Men (%)	46.3	45.2	45.2
Women (%)	53.7	54.8	54.8
Education (years)	12.6 ± 4.0	12.7 ± 4.0	–
BMI (kg/m^2^)	27.0 ± 4.9	26.8 ± 4.7^c^	26.9 ± 4.7^c^
WC (cm)	91.4 ± 13.7	90.6 ± 13.1^c^	92.8 ± 13.3^c^
Overweight, BMI ≥ 25 kg/m^2^ (%)	62.5	61.4^c^	62.3^c^
Obesity, BMI ≥ 30 kg/m^2^ (%)	21.7	19.9^c^	20.6^c^
Night sleep duration (hours)	7.3 ± 1.1	7.3 ± 1.0	–
Total physical activity (min/week)	743.7 ± 648.0	759.6 ± 652.4	–
Vigorous physical activity (min/week)	170.7 ± 249.1	176.4 ± 253.7	–
Depression^d^	9.9 ± 7.3	9.8 ± 7.3	–
Emotional eating^e^	1.9 ± 0.7	1.9 ± 0.7	–

*BMI* body mass index, *DILGOM* DIetary, Lifestyle and Genetic determinants of Obesity and Metabolic syndrome, *WC* waist circumference

^a^Ranges are given as number of participants with missing data (from 0.0 to 5.9%) varied between the study variables

^b^Mean ± SD (all such values)

^c^Calculated for participants with information on the respective variable from both study phases

^d^Sum score of 19 items from the Center for Epidemiological Studies – Depression Scale

^e^Mean score of 3 emotional eating items from the Three-Factor Eating Questionnaire-R18

Results from the confirmatory factor analysis supported the two-factor structure of the depression and emotional eating indicators. The two-factor model had an adequate fit with the data (Chi-Square = 48.4, df = 13, *p* < 0.001; CFI = 1.00; TLI = 1.00; RMSEA = 0.02; SRMR = 0.01) and each indicator loaded significantly (*P* < 0.001) on its respective latent factor with standardized factor loadings varying from 0.79 to 0.90 for emotional eating and from 0.45 to 0.91 for depression.

**Fig. 1:**
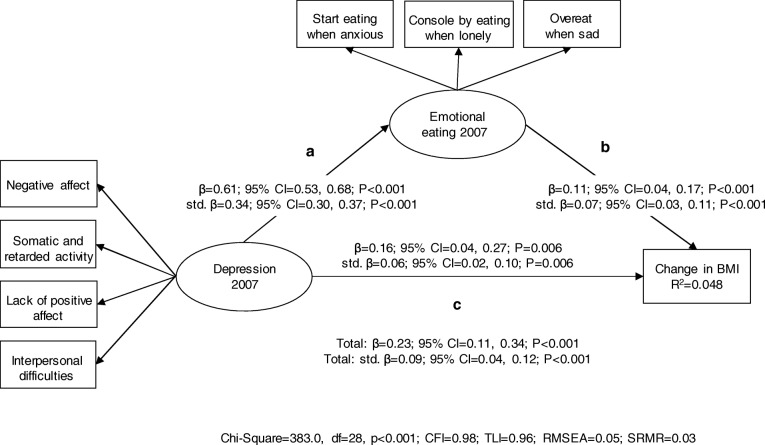
Results from the mediation model between depression, emotional eating and 7-year change in BMI (*n* = 4986). Depression and emotional eating were modelled as latent factors. Change in BMI was modelled by regressing the measurement at follow-up on the baseline measurement. The model was also adjusted for age and gender (not shown in Figure). Unstandardized and standardized regression coefficients (with 95% bias-corrected bootstrap confidence intervals) are represented on the arrows. Note. Total effect = c + ab. Indirect effect = ab. Indirect effect of depression on 7-year change in BMI: β = 0.068; 95% CI = 0.026, 0.105; *P* = 0.001 and std. β = 0.025; 95% CI = 0.009, 0.038; *P* = 0.001

**Fig. 2:**
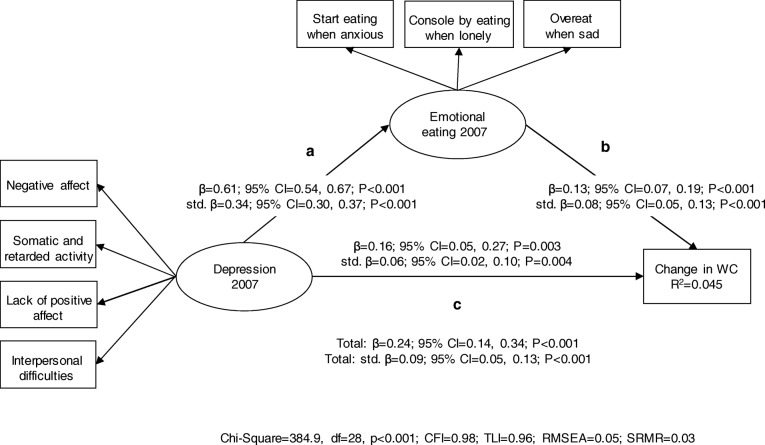
Results from the mediation model between depression, emotional eating and 7-year change in WC (*n* = 4985). Depression and emotional eating were modelled as latent factors. Change in WC was modelled by regressing the measurement at follow-up on the baseline measurement. The model was also adjusted for age and gender (not shown in Figure). Unstandardized and standardized regression coefficients (with 95% bias-corrected bootstrap confidence intervals) are represented on the arrows. Note. Total effect = c + ab. Indirect effect = ab. Indirect effect of depression on 7-year change in WC: β = 0.077; 95% CI = 0.041, 0.118; *P* < 0.001 and std. β = 0.028; 95% CI = 0.016, 0.043; *P* < 0.001

Figures 1 and 2 show that the mediation models between depression, emotional eating and 7-year change in BMI or WC fitted the data adequately. Depression and emotional eating were positively associated with each other and they both predicted higher 7-year increase in BMI and WC. The effects of depression on change in BMI (std. β = 0.025, *P* = 0.001 for the indirect effect) and WC (std. β = 0.028, *P* < 0.001 for the indirect effect) were mediated by emotional eating. These mediation models explained 4.8 and 4.5% of variance in BMI and WC change, respectively. Sensitivity analyses including only those participants (*n* = 1305–1310) with measured anthropometric data from both study phases produced comparable estimates with the exception that the effects of depression and emotional eating on WC change were not statistically significant at *P* < 0.05 level (see Additional files 2 and 3).

Gender did not moderate the associations of depression (*P* = 0.205–0.214 for the interaction terms) or emotional eating (*P* = 0.260–0.284 for the interaction terms) with change in BMI or WC (Table 2). However, while depression and emotional eating predicted higher BMI and WC gain in women, the estimates were non-significant in men. Emotional eating also mediated the effects of depression on change in BMI (β = 0.041, *P* = 0.190 in men and β = 0.085, *P* = 0.001 in women) and WC (β = 0.051, *P* = 0.110 in men and β = 0.093, *P* = 0.001 in women) only in women. The associations of depression with change in BMI (*P* < 0.001 for the interaction) and WC (*P* = 0.065 for the interaction) tended to vary according to age (Table 2). To interpret these interactions, we calculated simple slope tests at different values of the age moderator [49]: depression predicted higher BMI and WC gain at age 35 years and at age 50 years, but not at age 65 years.

**Table 2:** Gender and age as moderators of the associations between depression, emotional eating and 7-year change in adiposity indicators^a^

	Men	Women	Gender * latent factor interaction	35-year-olds	50-year-olds	65-year-olds	Age * latent factor interaction
	β (SE)^b^	β (SE)^b^	β (SE)	β (SE)^b^	β (SE)^b^	β (SE)^b^	β (SE)
Models for BMI							
EE 2007 ➔ BMI change	0.066 (0.050) *P* = 0.182	0.136 (0.037) *P* < 0.001	0.070 (0.062) *P* = 0.260	0.078 (0.051) *P* = 0.125	0.104 (0.031) *P* = 0.001	0.131 (0.040) *P* = 0.001	0.002 (0.002) *P* = 0.428
Depression 2007 ➔ BMI change	0.082 (0.085) *P* = 0.330	0.219 (0.067) *P* = 0.001	0.137 (0.108) *P* = 0.205	0.456 (0.092) *P* < 0.001	0.218 (0.054) *P* < 0.001	−0.020 (0.071) *P* = 0.780	−0.016 (0.004) *P* < 0.001
Models for WC							
EE 2007 ➔ WC change	0.082 (0.050) *P* = 0.101	0.149 (0.038) *P* < 0.001	0.067 (0.063) *P* = 0.284	0.027 (0.051) *P* = 0.595	0.108 (0.031) *P* < 0.001	0.190 (0.040) *P* < 0.001	0.005 (0.002) *P* = 0.016
Depression 2007 ➔ WC change	0.082 (0.084) *P* = 0.329	0.217 (0.068) *P* = 0.001	0.134 (0.108) *P* = 0.214	0.303 (0.093) *P* = 0.001	0.186 (0.054) *P* = 0.001	0.070 (0.072) *P* = 0.333	−0.008 (0.004) *P* = 0.065

*BMI* body mass index, *EE* emotional eating, *WC* waist circumference

^a^Models for gender were adjusted for age and models for age were adjusted for gender

^b^Estimates were derived by calculating simple slope tests at different values of the moderator [49]

Night sleep duration moderated the relationships of emotional eating with change in BMI (*P* = 0.007 for the interaction) and WC (*P* = 0.026 for the interaction) (Table 3). We again calculated simple slope tests at different values of the moderator to interpret these interactions: emotional eating predicted higher BMI and WC gain particularly at 6 h of sleep and at 7 h of sleep, while no such associations were observed at 9 h of sleep. Moreover, emotional eating mediated the effects of depression on change in BMI (e.g. β = 0.078, *P* = 0.049 for 6 h and β = − 0.002, *P* = 0.905 for 9 h) and WC (e.g. β = 0.075, *P* = 0.052 for 6 h and β = 0.009, *P* = 0.672 for 9 h) only in participants with shorter sleep duration. Total physical activity did not moderate the relationships of depression or emotional eating with change in BMI or WC (Table 3).

**Table 3:** Sleep and PA as moderators of the associations between depression, emotional eating and 7-year change in adiposity indicators^a^

	Night sleep duration (hours per night)					Total PA (minutes per week)			
	6 h	7 h	8 h	9 h	Sleep * latent factor interaction	95.7 min^b^	743.7 min^b^	1391.7 min^b^	PA * latent factor interaction
	β (SE)^c^	β (SE)^c^	β (SE)^c^	β (SE)^c^	β (SE)	β (SE)^c^	β (SE)^c^	β (SE)^c^	β (SE)
Models for BMI									
EE 2007 ➔ BMI change	0.212 (0.047) *P* < 0.001	0.139 (0.032) *P* < 0.001	0.066 (0.035) *P* = 0.059	−0.006 (0.054) *P* = 0.905	−0.073 (0.027) *P* = 0.007	0.106 (0.042) *P* = 0.012	0.108 (0.030) *P* < 0.001	0.110 (0.043) *P* = 0.010	0.000 (0.000) *P* = 0.937
Depression 2007 ➔ BMI change	0.073 (0.075) *P* = 0.333	0.128 (0.054) *P* = 0.019	0.184 (0.063) *P* = 0.003	0.239 (0.093) *P* = 0.010	0.056 (0.043) *P* = 0.192	0.267 (0.076) *P* < 0.001	0.174 (0.053) *P* = 0.001	0.082 (0.079) *P* = 0.300	0.000 (0.000) *P* = 0.097
Models for WC									
EE 2007 ➔ WC change	0.209 (0.047) *P* < 0.001	0.147 (0.032) *P* < 0.001	0.086 (0.036) *P* = 0.017	0.024 (0.056) *P* = 0.662	−0.062 (0.028) *P* = 0.026	0.133 (0.042) *P* = 0.002	0.125 (0.031) *P* < 0.001	0.116 (0.043) *P* = 0.006	0.000 (0.000) *P* = 0.778
Depression 2007 ➔ WC change	0.124 (0.075) *P* = 0.101	0.145 (0.055) *P* = 0.008	0.166 (0.064) *P* = 0.009	0.187 (0.094) *P* = 0.045	0.021 (0.043) *P* = 0.622	0.191 (0.077) *P* = 0.013	0.172 (0.054) *P* = 0.001	0.154 (0.078) *P* = 0.049	0.000 (0.000) *P* = 0.737

*BMI* body mass index, *EE* emotional eating, *PA* physical activity, *WC* waist circumference

^a^Models were adjusted for age and gender

^b^1 SD below the mean (95.7 min/week), mean (743.7 min/week), and 1 SD above the mean (1391.7 min/week)

^c^Estimates were derived by calculating simple slope tests at different values of the moderator [49]

Finally, the association between depression and emotional eating did not vary according to gender (*P* = 0.970–0.981 for the interactions terms), age (*P* = 0.766–0.782, respectively), night sleep duration (*P* = 0.120–0.131, respectively) or physical activity (*P* = 0.072–0.075, respectively) in any of the tested models.

## Discussion

To our best knowledge, this is the first study to examine the mediation effect of emotional eating between depression and long-term weight changes in the context of gender, age, night sleep duration and physical activity patterns. There are two main findings: Firstly, we found that eating in response to negative emotions mediated the positive associations between depression and increase in BMI and WC over 7 years – a finding providing support for the hypothesis that emotional eating is one behavioural mechanism between depression and subsequent development of obesity and abdominal obesity. Secondly, we observed that night sleep duration moderated the associations of emotional eating: individuals with higher emotional eating and shorter sleep duration were particularly vulnerable to BMI and WC gain.

Our results regarding the mediation effect of emotional eating are consistent with two prospective studies conducted in Dutch parents [18] and mid-life US adults [19] with self-reported anthropometrics (BMI and a composite of BMI and WC, respectively) and confirm our cross-sectional results in the baseline data of the DILGOM study [5]. The present prospective research extends observations from the Dutch and US samples by having also measured information on obesity (BMI) and abdominal obesity (WC) indicators, analyzing them as separate outcomes and testing several moderators (i.e. gender, age, sleep and physical activity) simultaneously. In the Dutch and US samples, emotional eating acted as a mediator between depression and risk of developing obesity only in women. Although gender did not have statistically significant moderator effects in our study, we found a consistent trend resembling this gender difference: the direct and indirect effects of depression and emotional eating on BMI and WC gain were more pronounced in women than in men (and significant only in women). The stronger effects in women are likely to be linked to their higher susceptibility to engage in emotional eating [5, 16, 26] and experience symptoms of depression [54]. Sex differences in physiological stress response could also bear relevance. The typical physiological response is hyper-activation of the hypothalamic-pituitary-adrenal axis and decreased appetite, while adult women often show lower hypothalamic-pituitary-adrenal axis and autonomic stress responses than men of same age [55]. Evidence has further suggested a role for blunted rather than enhanced cortisol response to stress in increased food intake of high emotional eaters [56], binge eaters [57] or chronically highly stressed [58].

In accordance with two earlier studies examining the interplay between emotional eating and sleep duration in the development of obesity, we found that the positive associations of emotional eating with BMI and WC gain were stronger in the short sleepers (e.g. 6 h per night) than in the long sleepers (e.g. 9 h per night). Emotional eating consequently mediated the link between depression and weight gain primarily in those sleeping fewer hours per night. The fact that a similar moderation effect has now been detected in three independent samples of French Canadian adults [33], Dutch employees [29] and Finnish adults builds confidence on the robustness of this observation. Evidence is also emerging that sleep restriction enhances brain neuronal activation in response to unhealthy food stimuli compared with non-restricted sleep [59] – suggesting that short sleep duration is a type of stressor that is especially likely to induce increased food intake in emotional eaters. It is though noteworthy that short sleepers are a heterogeneous group involving at least three types of individuals: those for whom short sleep schedule represents their natural way of functioning, those who reduce their sleep time to meet other demands of daily life, and those who have sleeping difficulties [60]. Thus, short sleep is likely to be a source of stress or a marker of perceived stress only for the latter two types of people. Yet, as a whole, our findings highlight that individuals with a combination of shorter night sleep duration and higher degree of emotional eating may require tailored approaches in weight management programs.

In contrast to our expectations, we did not find evidence that the level of total physical activity would moderate the relationships between depression, emotional eating and change in BMI and WC. However, consistent with previous observations [25, 26] individuals with higher levels of vigorous and total physical activity scored slightly lower on emotional eating. Regarding the lack of the moderator effect, it is possible that engaging in activities of vigorous intensity is particularly relevant: some observational studies (though not all) have reported stronger associations between vigorous physical activity and decreased likelihood of depression as compared to the associations involving moderate activities [61]. In the study of the Dutch employees, particularly strenuous physical activity (running, working out) moderated the association of emotional eating with BMI change [34]. We repeated the moderator analyses with dichotomous (42% non-vigorous vs. 58% vigorous) and continuous vigorous physical activity scores, but again did not detect statistically significant interactions (*P* = 0.194–0.971 for the interactions involving emotional eating and *P* = 0.106–0.771 for the interactions involving depression). However, this could be at least partly explained by present participants’ relatively low levels of vigorous activities.

Because of the large age range (between 25 and 74 years at baseline) in our study, we additionally examined whether the associations varied across age groups. The results suggested that symptoms of depression predicted BMI and WC gain especially in younger participants. Age-related changes in body composition and weight offer one potential explanation for this observation. For instance, aging is known to lead to decreases in muscle mass [62]. In the present sample, WC increased more in 25–34-year-olds than in 65–74-year-olds and BMI even slightly decreased in 65–74-year-olds over 7 years. It is therefore possible that such age-related patterns have obscured the effects in older adults.

Individuals may engage in emotional eating to cope with stress and other negative emotions, but in the long-term it is often a maladaptive emotion regulation strategy. Besides that emotional eating may lead to less healthy food intake patterns and subsequent weight gain, it is unlikely to result on long-term improvements in mood – i.e. intake of palatable food has shown to improve experimentally induced negative mood state immediately, but the effect tends to be short-term and is easily followed by other negative emotions (e.g. feelings of guilt) especially in dieters [63, 64]. Individuals with a high susceptibility to emotional eating might therefore benefit from interventions that teach emotion regulation skills as is done in dialectical behaviour therapy [65] or that incorporate mindfulness training [66]. The present findings also imply that future randomized controlled trials could test whether extending sleep is a viable strategy to prevent weight gain and promote healthier food intake in emotional eaters. Interestingly, a recent pilot study in habitually short sleepers (with no information on emotional eating) demonstrated that sleep extension was feasible and led to decreased intake of free sugars [28].

A particular strength of the present study is that it was based on a large population-based sample with 7-year follow-up on both BMI and WC. The wealth of both measured and self-reported health-related information and the prospective design allowed us to provide novel insights on depression and emotional eating as risk factors for (abdominal) obesity. However, certain limitations need to be taken into account while interpreting the results. Firstly, although the sample was initially randomly derived from the Finnish population register, there were non-participants as in all observational studies. We detected small differences between participants and non-participants at follow-up in terms of baseline age, gender, BMI and WC. Despite that we used FIML to handle missing data, which has shown to produce less biased estimates than conventional techniques, such as listwise deletion [50, 51], our observations could still generalize less well to younger men and individuals with higher initial weight. Secondly, although measured anthropometric data were available for all participants at baseline, two-thirds of the participants self-reported their height, weight and WC at follow-up with measured data available for one-third [38]. Nonetheless, sensitivity analyses excluding those with self-reported anthropometrics at follow-up supported our findings by producing fairly comparable point estimates. Thirdly, the widely used CES-D scale and TFEQ-R18 have also some restrictions: the former does not yield information on clinical depression, while the latter contains only three items to measure emotional eating. Fourthly, night sleep duration and physical activity tested as moderators in this study could alternatively be hypothesized to mediate the depression – obesity link. For that reason, we conducted a final set of mediation models testing these hypotheses, but there was no consistent evidence for the mediation effect of physical activity (*P* = 0.529 for indirect effect on BMI and *P* = 0.684 for indirect effect on WC) or sleep duration (*P* = 0.056 and *P* = 0.682, respectively) in line with a recent 4-year prospective cohort study [67]. Finally, it should be noted that the tested mediation models including depression, emotional eating, gender and age as predictors explained only around 5% of variance in change in BMI and WC, which highlights the well-recognized fact that long-term weight changes are influenced by myriad of factors.

## Conclusions

The present findings highlight the interplay between depression, emotional eating and short night sleep duration in influencing subsequent development of obesity and abdominal obesity. Future research should test the clinical significance of our observations by tailoring weight management programs according to these characteristics.

## Additional files

Additional file 1: Pearson’s correlation coefficients between the main study variables. (DOCX 14 kb)

Additional file 2: Results from sensitivity analysis including only those participants (*n* = 1310) whose height and weight were measured at baseline and follow-up: the mediation model between depression, emotional eating and 7-year change in BMI. (DOCX 36 kb)

Additional file 3: Results from sensitivity analysis including only those participants (*n* = 1305) whose WC was measured at baseline and follow-up: the mediation model between depression, emotional eating and 7-year change in WC. (DOCX 37 kb)

## Abbreviations

BMI: Body mass index
CES-D: Center for Epidemiological Studies – Depression
CFI: Comparative Fit Index
CI: Confidence interval
DILGOM: DIetary, Lifestyle and Genetic determinants of Obesity and Metabolic syndrome
EE: Emotional eating
FIML: Full Information Maximum Likelihood
IPAQ-SF: International Physical Activity Questionnaire - Short Form
PA: Physical activity
RMSEA: Root Mean Square Error of Approximation
SD: Standard deviation
SE: Standard error
SRMR: Standardized Root Mean Square Residual
TFEQ-R18: Three-Factor Eating Questionnaire-R18
TLI: Tucker-Lewis Index
US: United States
WC: Waist circumference
