# Supplementary material for: Depression, emotional eating and long-term weight changes: a population-based prospective study
Source: Int J Behav Nutr Phys Act. 2019 Mar 20;16:28. doi: 10.1186/s12966-019-0791-8 (PMC6427874; doi:10.1186/s12966-019-0791-8)
Supplement: Supplementary file 1 — Pearson’s correlation coefficients between the main study variables. (DOCX 14 kb) [file 12966_2019_791_MOESM1_ESM.docx]

Additional file 1. Pearson’s correlation coefficients between the main study variables^1^

|  | 1 | 2 | 3 | 4 | 5 | 6 | 7 | 8 | 9 | 10 | 11 | 12 |
| --- | --- | --- | --- | --- | --- | --- | --- | --- | --- | --- | --- | --- |
| 1. Age 2007 | 1.00 |  |  |  |  |  |  |  |  |  |  |  |
| 2. BMI 2007 | 0.20*** | 1.00 |  |  |  |  |  |  |  |  |  |  |
| 3. BMI 2014 | 0.10*** | 0.90*** | 1.00 |  |  |  |  |  |  |  |  |  |
| 4. WC 2007 | 0.26*** | 0.87*** | 0.77*** | 1.00 |  |  |  |  |  |  |  |  |
| 5. WC 2014 | 0.16*** | 0.74*** | 0.82*** | 0.86*** | 1.00 |  |  |  |  |  |  |  |
| 6. BMI change | -0.18*** | 0.07*** | 0.50*** | 0.04** | 0.39*** | 1.00 |  |  |  |  |  |  |
| 7. WC change | -0.11*** | 0.07*** | 0.37*** | 0.08*** | 0.58*** | 0.69*** | 1.00 |  |  |  |  |  |
| 8. Night sleep duration 2007 | -0.04** | -0.03* | -0.03 | -0.05*** | -0.05** | -0.04* | -0.03 | 1.00 |  |  |  |  |
| 9. Total PA 2007 | 0.12*** | -0.04** | -0.03* | -0.05** | -0.03 | -0.02 | -0.01 | -0.04** | 1.00 |  |  |  |
| 10. Vigorous PA 2007 | 0.07*** | -0.02 | -0.02 | 0.01 | 0.04* | -0.01 | 0.03 | -0.03* | 0.66*** | 1.00 |  |  |
| 11. Depression 2007^2^ | 0.11*** | 0.11*** | 0.12*** | 0.10*** | 0.11*** | 0.04* | 0.04* | -0.11*** | -0.04** | -0.05** | 1.00 |  |
| 12. Emotional eating 2007^3^ | -0.07*** | 0.26*** | 0.27*** | 0.14*** | 0.15*** | 0.10*** | 0.06** | 0.01 | -0.07*** | -0.09*** | 0.29*** | 1.00 |

BMI = body mass index, PA = physical activity, WC = waist circumference.

^1^N=3574-5024 (number of participants with missing data varied between the study variables).

^2^Sum score of 19 items from the Center for Epidemiological Studies – Depression Scale.

^3^Mean score of 3 emotional eating items from the Three-Factor Eating Questionnaire-R18.

***P<0.001, **P<0.01, *P<0.05.
