# Supplementary material for: Depression, emotional eating and long-term weight changes: a population-based prospective study
Source: Int J Behav Nutr Phys Act. 2019 Mar 20;16:28. doi: 10.1186/s12966-019-0791-8 (PMC6427874; doi:10.1186/s12966-019-0791-8)
Supplement: Supplementary file 2 — Results from sensitivity analysis including only those participants (n = 1310) whose height and weight were measured at baseline and follow-up: the mediation model between depression, emotional eating and 7-year change in BMI. (DOCX 36 kb) [file 12966_2019_791_MOESM2_ESM.docx]

Additional file 2. Results from sensitivity analysis including only those participants (n=1310) whose height and weight were measured at baseline and follow-up: the mediation model between depression, emotional eating and 7-year change in BMI.

Note. Depression and emotional eating were modelled as latent factors. Change in BMI was modelled by regressing the measurement at follow-up on the baseline measurement. The model was also adjusted for age and gender (not shown in Figure). Unstandardized and standardized regression coefficients (with 95% bias-corrected bootstrap confidence intervals) are represented on the arrows. Indirect effect of depression on 7-year change in BMI: β=0.062; 95% CI=0.010, 0.118; P=0.028 and std. β=0.024; 95% CI=0.004, 0.045; P=0.026.
