# Supplementary material for: Depression, emotional eating and long-term weight changes: a population-based prospective study
Source: Int J Behav Nutr Phys Act. 2019 Mar 20;16:28. doi: 10.1186/s12966-019-0791-8 (PMC6427874; doi:10.1186/s12966-019-0791-8)
Supplement: Supplementary file 3 — Results from sensitivity analysis including only those participants (n = 1305) whose WC was measured at baseline and follow-up: the mediation model between depression, emotional eating and 7-year change in WC. (DOCX 37 kb) [file 12966_2019_791_MOESM3_ESM.docx]

Additional file 3. Results from sensitivity analysis including only those participants (n=1305) whose WC was measured at baseline and follow-up: the mediation model between depression, emotional eating and 7-year change in WC.

Note. Depression and emotional eating were modelled as latent factors. Change in WC was modelled by regressing the measurement at follow-up on the baseline measurement. The model was also adjusted for age and gender (not shown in Figure). Unstandardized and standardized regression coefficients (with 95% bias-corrected bootstrap confidence intervals) are represented on the arrows. Indirect effect of depression on 7-year change in WC: β=0.040; 95% CI=-0.012, 0.093; P=0.138 and std. β=0.016; 95% CI=-0.004, 0.037; P=0.137.
